# Inhibitory checkpoint molecule mRNA expression in canine soft tissue sarcoma

**DOI:** 10.1111/vco.12934

**Published:** 2023-09-07

**Authors:** Valentina Beatriz Stevenson, Erwin Kristobal Gudenschwager-Basso, Shawna Klahn, Tanya LeRoith, William R. Huckle

**Affiliations:** 1 Department of Biomedical Sciences & Pathobiology, Virginia-Maryland College of Veterinary Medicine, Virginia Tech, Blacksburg, Virginia, USA; 2 Department of Small Animal Clinical Sciences, Virginia-Maryland College of Veterinary Medicine, Virginia Tech, Blacksburg, Virginia, USA

**Keywords:** canine, PD-1, PD-L1, PD-L2, soft tissue sarcoma, TILs

## Abstract

Canine soft tissue sarcomas (STS) are common neoplasms and considered immune deserts. Tumour infiltrating lymphocytes are sparse in STS and, when present, tend to organize around blood vessels or at the periphery of the neoplasm. This pattern is associated with an immunosuppressive tumour microenvironment linked to overexpression of molecules of the PD-axis. PD-1, PD-L1 and PD-L2 expression correlates with malignancy and poor prognosis in other neoplasms in humans and dogs, but little is known about their role in canine STS, their relationship to tumour grade, and how different therapies affect expression. The objective of this study was to evaluate the expression of checkpoint molecules across STS tumour grades and after tumour ablation treatment. Gene expression analysis was performed by reverse-transcriptase real-time quantitative PCR in soft tissue sarcomas that underwent histotripsy and from histologic specimens of STS from the Virginia Tech Animal Laboratory Services archives. The expression of PD-1, PD-L1 and PD-L2 was detected in untreated STS tissue representing grades 1, 2, and 3. Numerically decreased expression of all markers was observed in tissue sampled from the treatment interface relative to untreated areas of the tumour. The relatively lower expression of these checkpoint molecules at the periphery of the treated area may be related to liquefactive necrosis induced by the histotripsy treatment, and would potentially allow TILs to infiltrate the tumour. Relative increases of these checkpoint molecules in tumours of a higher grade and alongside immune cell infiltration are consistent with previous reports that associate their expression with malignancy.

## 1 ∣ INTRODUCTION

After many years of relying on conventional approaches of surgery, chemotherapy, and radiation to treat cancer, the introduction of immunotherapies has revolutionized the field, promising numerous advantages for people^1^ and dogs.^2^ Among the most successful immunotherapies in humans are those that aim to block the interaction between Programmed Death-1 (PD-1), a suppressive immunoreceptor expressed in exhausted T cells, and its ligands PD-L1 and PD-L2.^3-5^ PD-L1 is frequently expressed at high levels on malignant tumour cells and, upon the binding of this ligand with the PD-1 receptor on T cells, leads to immunotolerance, allowing tumour progression and increased rate of metastasis.^4,6-9^ Over the last several years, attempts made to develop similar therapies for canine melanoma have produced reports of decreased tumour size, prolonged survival times, and complete remission.^10,11^ Efficacy of immunotherapies in humans is strongly correlated with the immune profile of the tumour microenvironment (TME).^8,12^ Better outcomes are usually associated with “hot” tumours, defined by increased levels of T cell infiltration and immune stimulatory cytokines, while these therapies have less efficacy in “cold” tumours, which maintain an immunoinhibitory TME, with low levels of T cell infiltration.^2,12,13^

Soft tissue sarcomas (STS) are common in dogs, representing up to 15% of all cutaneous or subcutaneous tumours.^14^ They are a diverse group of neoplasms of mesenchymal origin with similar characteristics: resection is difficult, the rate of recurrence is high, and metastasis can develop in up to 30% of the cases. Factors that contribute to poorer prognosis are a higher mitotic count, increased pleomorphism, areas of necrosis, and a tumour size larger than 5 cm.^14,15^ STS are usually graded according to guidelines proposed by Trojani et al. and modified by Dennis et al. Briefly, a score from 1 to 3 is given based on differentiation of the neoplasm, another score from 1 to 3 based on mitotic count, and a score from 0 to 2 based on degree of necrosis within the neoplasm.^16,17^

STSs have long been considered immune deserts and thought to be poor candidates for immune therapies.^18-20^ However, recent studies report that infiltration of Tumour Infiltrating Lymphocytes (TILs) depends on tumour type and that highly mutated tumours with elevated Copy Number Alterations have a higher immunogenicity.^19,21,22^ Expression of PD-L1 has already been described in human STS, where an association with poor prognosis and shorter survival times was reported.^23-26^ In contrast, evaluation of the expression of this checkpoint molecule in canine cancers is scarce.^11,27,28^ In STS there is only one report, in which PD-L1 immunopositivity was noted in two of eleven hemangiosarcomas, six of eight malignant fibrous histiocytomas, four of eleven malignant nerve sheath tumours, four of ten hemangiopericytomas, and four of six fibrosarcomas.^29^ PD-L1 has also been reported in serum of a group of nine dogs with malignant mesenchymal tumours whose diagnosis included osteosarcoma, liposarcoma, cutaneous hemangiosarcoma, fibrosarcoma, and leiomyosarcoma.^30^

Regardless of the immune status of the tumour, most therapies aim not only to destroy cancer cells, but also to release tumour antigens to the TME to promote their recognition by the immune system, both at the primary site and in circulation, collectively known as abscopal response.^31-34^ This approach has been successful in human STS, as increasing the immunogenicity of this rather “cold” tumour amplifies T cell recognition and later tumour destruction, giving combined immunotherapies advantage over monotherapies.^8,35,36^

Alternative local tumour ablation therapies have been developed over the last six years. These therapies aim for a less invasive technique, targeting cancer cells and sparing as many healthy cells as possible.^37,38^ Tumour ablation therapies include radiofrequency ablation, microwave ablation, laser, high-intensity focused ultrasound, cryoablation, irreversible electroporation, and histotripsy.^38^ The first six of these modalities rely on extreme temperatures to ablate the tumour tissue, whereas histotripsy is among the first non-ionizing and non-thermal ablation image-guided therapies. Microsecond pulses of soundwave pressure cavitate microbubbles within the tissue, generating mechanical stress and liquifying neighbouring cancer cells with precise margins^34,39^ thus sparing critical normal structures such as nerves and large blood vessels. The homogenized and liquified tissue contains native tumour antigens with retained antigenicity, which may promote an abscopal or distal immune response and can inhibit the process of metastasis by both increasing recognition of antigens by dendritic cells and Damage Associated Molecular Patterns by Pattern Recognition Receptors including Toll-like receptors that feed the cytokine cascade that promote inflammation.^33,34,37,40^ After the initial response by the innate immune system, CD8+ cytotoxic and CD4+ helper T cells are activated and can travel to the tumour site via the circulation to interact with the tumour cells.^37,39,41^

Although the immune response after histotripsy has been recently described in canine cancers, reports are still limited,^42-44^ especially in STS for which a sole report exists.^40^ The adaptive response to histotripsy is currently under investigation, and the expression of checkpoint molecules from the PD-axis after therapy is relatively unexplored. With the evolution of immunotherapies in human medicine, it is imperative that more efforts are made to gain insight in the treatment of veterinary cancer patients and to better understand how immunotherapies could bring answers either as sole therapy or as adjuvants to tumour ablation therapies. To our knowledge, this is the first report that describes the expression of checkpoint molecules in canine STS.

## 2 ∣ MATERIALS AND METHODS

### 2.1 ∣ Tissue samples

Cases selected for this study consist of 2 different groups. One group includes 29 cases selected from the archives of Virginia Tech Animal Laboratory Services (ViTALS) with the diagnosis of soft tissue sarcoma, representing several of the recognized STS histotypes (Supplemental Table 1). These tumour biopsies were from different privately owned dogs, submitted for routine histopathological evaluation. All grade 1 and 2 cases were from 2021. Grade 3 cases were from 2019 (3), 2020 (1), and 2021 (5). The diagnosis of soft tissue sarcoma was further classified as grade 1, 2, and 3 according to guidelines proposed by Trojani et al. and modified by Dennis et al. 2011.^16,17^ Briefly, a score from 1 to 3 was assigned based on differentiation of the neoplasm, another score from 1 to 3 was assigned based on mitotic count, and a score from 0 to 2 was assigned based on degree of necrosis within the neoplasm. Lymphovascular invasion was morphologically assessed. Following this classification system, 10 cases were assigned as grade 1, 10 as grade 2, and 9 as grade 3.

The second group consisted of cases from a clinical study performed by Ruger et al.,^40^ that aimed to determine the safety and feasibility of histotripsy therapy in ten canine patients with soft tissue sarcoma. The histotripsy inclusion criteria were patients with a cytological or histological diagnosis of soft tissue sarcoma and had received no prior treatment. Recurrent tumours were not excluded. Excisional biopsies were taken four to six days after histotripsy treatment and submitted for evaluation by a veterinary pathologist with extensive experience evaluating ablated tumour tissue, and tissue sections were stored as formalin-fixed paraffin-embedded (FFPE) blocks. For the current study, tissue sections collected from the area of the treatment interface (Supplemental Figure 1) and from distal untreated areas of the tumour for each patient were selected for analysis.

### 2.2 ∣ Real-time quantitative polymerase chain reaction (RT-qPCR)

Ten scrolls of 10 um in thickness each were serially cut from FFPE blocks from each of the 49 cases. RNA extraction and cDNA synthesis were performed using commercially available kits (Qiagen RNA extraction kit, and Life Technologies High Capacity cDNA kit, respectively) according to manufacturer's protocols. RT-qPCR analysis was performed using previously validated primers and probes spanning variant-conserved exon-exon junctions in triplicate 20 ul reactions as described by Stevenson et al.^27^ For PD-L1 and PD-L2 targets, validated primer and TagMan probe concentrations were 0.3 uM and 0.2 uM, respectively; primers and probe sets for canine PD-1 and the internal reference marker 18S rRNA obtained from ThermoFisher (cat. nos. 4 351 372 and 4333760F, respectively) were used as recommended by the vendor. TaqMan Fast Universal PCR Master Mix (ThermoFisher cat. no. 4366073) was used for all reactions. Negative controls that either omitted added template or contained non-reverse transcribed RNA were included to confirm that qPCR signals observed were attributable to the presence of cDNA.

For each sample, mean Ct values for each target were first normalized internally to 18S rRNA expression (yielding ΔCt), then compared to the same target's mean normalized expression in Grade 1 tumours (first study described above) or Untreated tumours (second study), yielding ΔΔCt. For display, results were calculated as a fold change (2^−ΔΔCt^) relative to mean expression in the respective group used as control.

### 2.3 ∣ Immunohistochemistry

Immunohistochemical analysis was performed on all 10 cases from the histotripsy clinical study, both at the treatment interface and untreated tissue sections, using antibodies against CD3 (pan T-cell marker) and FoxP3 (a regulatory T-cell marker). Sections of 5 um from each FFPE tissue section were deparaffinized and stained with a monoclonal antibody for CD3 (Dako clone A0452; 1:100), and anti-HuFoxP3 (eBio clone 7979; 1:100).^45^ The Universal Alkaline Phosphatase Red Detection kit (Ventana UltraView) was used as secondary antibody, and visualization of the antibody was obtained with Fast Red chromogen incubation. Slides were processed on an automated Ventana Benchmark XT using standard protocols at Virginia Tech Animal Laboratory Services, an AAVLD-accredited veterinary diagnostic laboratory. Incubation conditions for immunohistochemical analysis are detailed in the Supplemental Material. Positive controls for each cell marker were tissues from colon and lymph nodes. Sections of soft tissue sarcoma tissue stained without addition of primary antibody served as negative controls. Haematoxylin was used as a counterstain. Assessment and characterization of the 16 tissue sections were performed in a blinded fashion by a single board-certified veterinary pathologist counting individual immunopositive cells infiltrating the tumour averaged over 10 high-power fields (HPF; 2.37 mm^2^) in each tissue section.

### 2.4 ∣ Statistical analysis

Normality of distribution of relative expression data for individual checkpoint molecules (as 2^−ΔΔCt^ values) was assessed by Shapiro–Wilk test. Where data were not normally distributed, comparisons between groups were made using either a non-parametric Kruskal-Wallis test, with Dunn's test used to correct for multiple comparisons, or a two-tailed unpaired non-parametric Kolmogorov- Smirnov test. Two-sided t tests were used to compare checkpoint molecule expression in untreated and ultrasound-treated tumours. The relationship between the abundance of CD3+ and FoxP3+ and relative gene expression of each check point molecule was assessed using least-squares linear regression, with statistical significance of non-zero slopes calculated using analysis of variance. The correlation coefficient (r) of the regression was used to estimate the degree of association between the two variables. Results were considered statistically significant when *p* values < 0.05. All statistical analyses were performed using Prism 9.4 (GraphPad software).

## 3 ∣ RESULTS

### 3.1 ∣ Expression of inhibitory checkpoint molecules in soft tissue sarcoma of grades 1, 2 and 3

RT-qPCR analysis was performed targeting *PD-1*, *PD-L1*, and *PD-L2* using total RNA extracted from 29 FFPE soft tissue sarcoma tissue blocks. Results for each specimen are expressed as checkpoint molecule mRNA normalized internally to 18S rRNA and relative to mean normalized expression in Grade 1 tissue sections (2^−ΔΔCt^).

**FIGURE 1:**
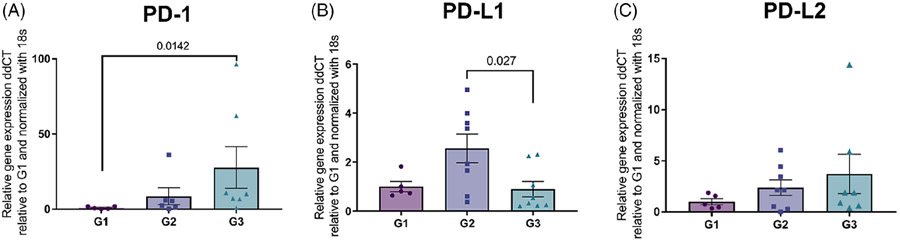
Relative expression (mean +/− SEM) of PD-1 (A), PD-L1 (B), and PD-L2 (C) mRNA in canine soft tissue sarcoma of Grade 1, 2, and 3, as assessed by Kruskal-Wallis test. Each column represents a different tumour grade, and each point represent an individual case. Data for each target and STS case were internally normalized to 18S rRNA expression and are shown relative to mean normalized expression in Grade 1 tumours.

The patterns of expression of checkpoint molecule mRNAs in soft tissue sarcomas of increasing grade were variable (Figure 1). Mean expression of PD-1 increased with tumour grade, reaching at Grade 3 statistical significance relative to Grade 1 (Figure 1A; *p* = 0.0142). Interestingly, PD-L1 relative expression was statistically significantly higher in Grade 2 when compared to Grade 3 (*p* = 0.027), but expression was not statistically significantly different between Grades 1 and 3 (Figure 1B). Expression of PD-1 and PD-L2 mRNAs increased numerically as tumour grade increased, but these changes did not reach statistical significance.

**FIGURE 2:**
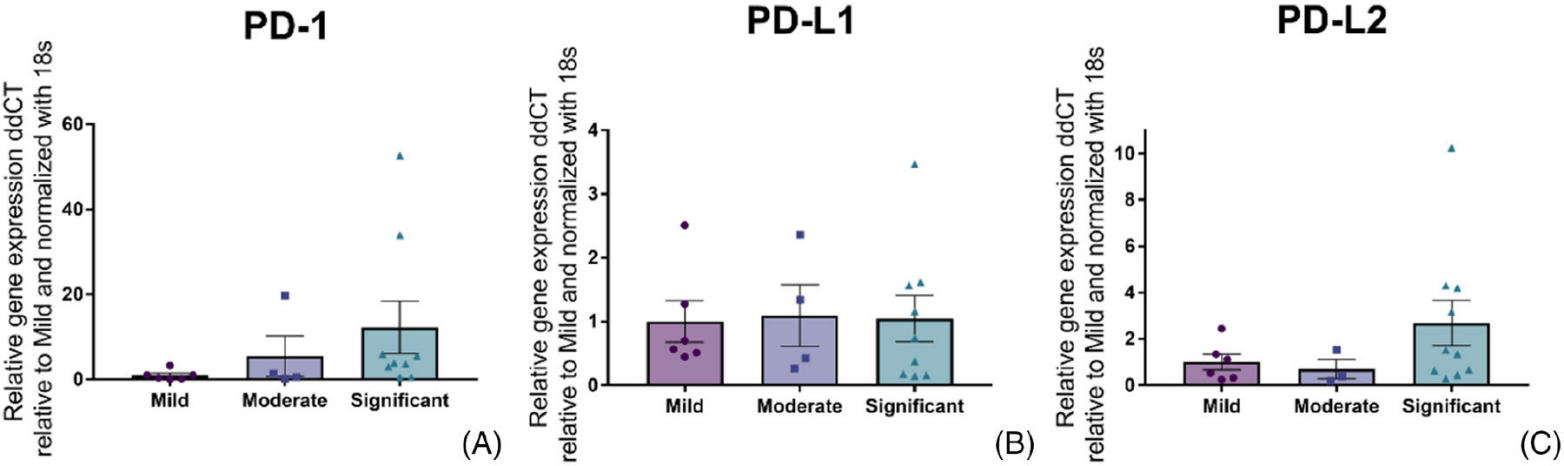
Relative expression (mean +/− SEM) of PD-1 (A), PD-L1 (B), and PD-L2 (C) mRNA in canine soft tissue sarcoma with mild, moderate, and significant degree of undifferentiation. PD-1 and PD-L2 expression is numerically higher in soft tissue sarcomas with more significant undifferentiation. PD-L1 expression remained similar regardless differentiation grade of the soft tissue sarcoma.

To evaluate the relationship between anaplasia of the soft tissue sarcomas and the expression of the inhibitory checkpoint molecules of interest, all tissue sections were reclassified based on degree of differentiation of the tumour. All sections were evaluated based only on degree of differentiation using the suggested score system by Dennis et al.^17^ Samples were given the score 1 = Mild when the cells largely resembled normal tissue; score 2 = Moderate when there was an identifiable pattern of soft tissue, but only modest resemblance to normal tissue; and score 3 = Significant when the tumour cells were undifferentiated. After this classification, these scores were compared to their expression of PD-1, PD-L1 and PD-L2. The measured expression of PD-1 and PD-L2 was numerically higher in tumours with a higher degree of differentiation, whereas PD-L1 gene expression remained numerically similar regardless of the degree of differentiation (Figure 2). No statistically significantly different comparisons were observed (Kruskal-Wallis).

### 3.2 ∣ Expression of inhibitory checkpoint molecules in soft tissue sarcomas from a clinical trial using histotripsy

**FIGURE 3:**
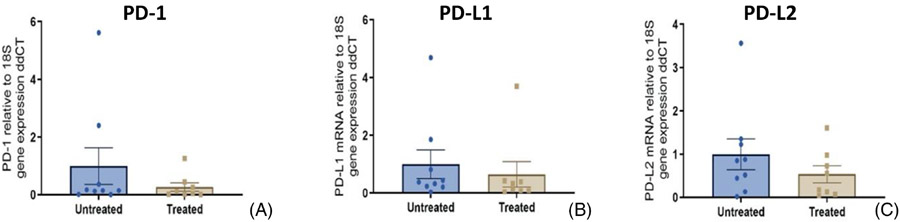
Relative expression (mean +/− SEM) of PD-1 (A), PD-L1 (B), and PD-L2 (C) mRNA in soft tissue sarcomas treated with histotripsy, showing comparison between PD-axis gene expression in tissue sections from the treatment interface, and untreated areas of the neoplasm.

Measurement of PD-1, PD-L1, and PD-L2 gene expression was performed as described above using RNA extracted from 20 tissue sections, two per individual STS patient: 10 from treatment interface areas and 10 from untreated areas of soft tissue sarcomas that had undergone histotripsy treatment (Figure 3). Each specimen's results are calculated as checkpoint molecule mRNA normalized internally to 18S rRNA, then as relative to the mean normalized expression in the untreated tissue sections (2^−ΔΔCt^). All 3 checkpoint molecules measured expression were numerically lower in the treatment interface when compared to the untreated areas of the tumour, but did not reach statistical significance (Kolmogorov–Smirnov test).

### 3.3 ∣ Immunohistochemistry

**FIGURE 4:**
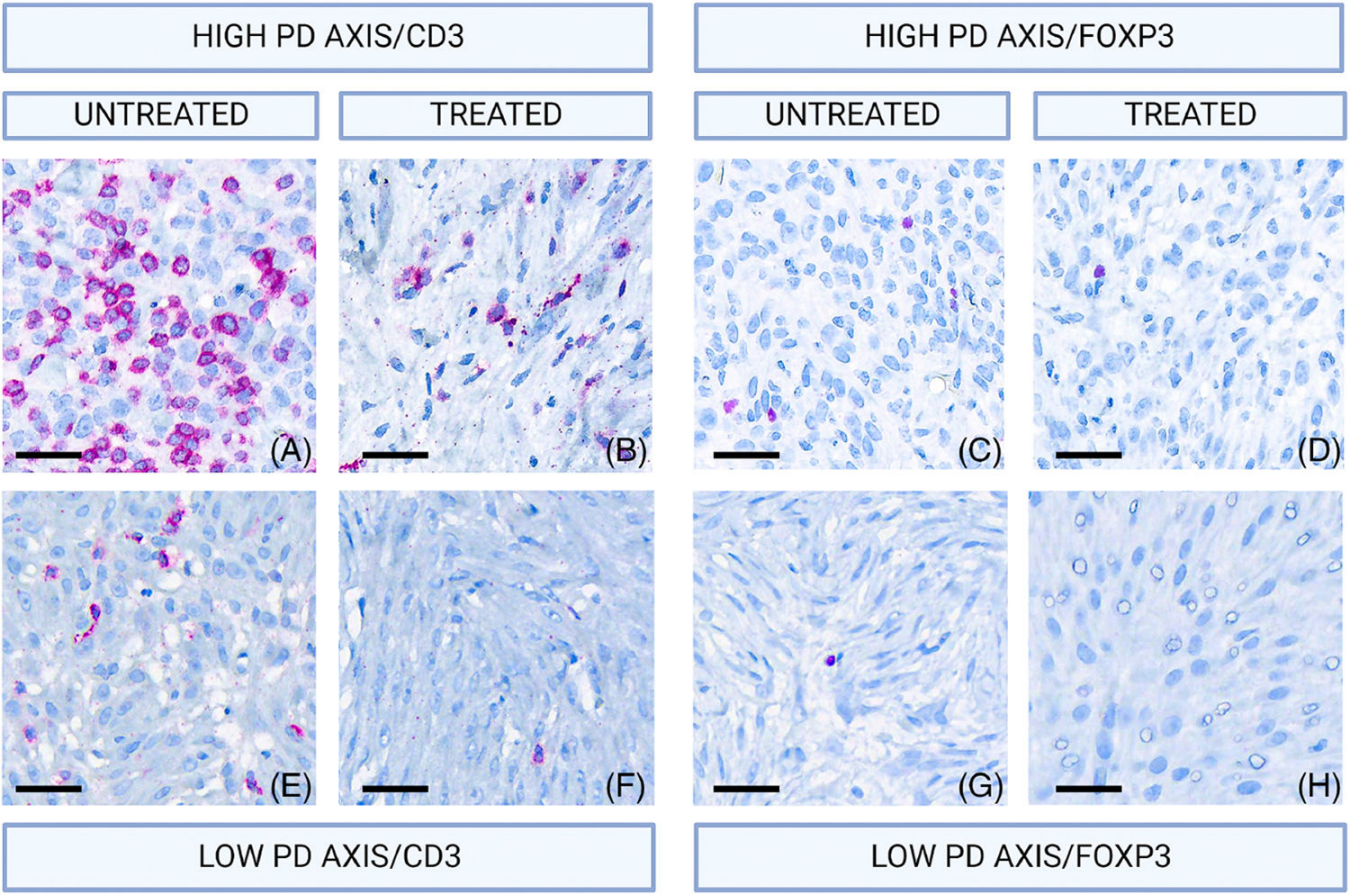
Micrographs from IHC analysis (40× magnification, Fast Red chromogen with haematoxylin counterstain). Images on the top row (A–D) show a patient with high expression of the PD-axis and high infiltration of T lymphocytes. Images on the bottom row (F–I) show a patient with low expression of the PD-axis mRNAs and low infiltration of T lymphocytes. CD3+ cells are highlighted in images A, B, F, G, where a higher infiltration of CD3+ positive cells are seen in an untreated tumour section (panel A). Similarly, Foxp3+ cells are highlighted in images C, D, H, I, with higher numbers of Foxp3+ cells observed in image C.

In order to evaluate the effects of the histotripsy therapy on the presence of TILs in the local TME, immunohistochemistry was performed to identify T lymphocytes using CD3 as a marker and T regulatory cells using Foxp3 (Figure 4).

**FIGURE 5:**
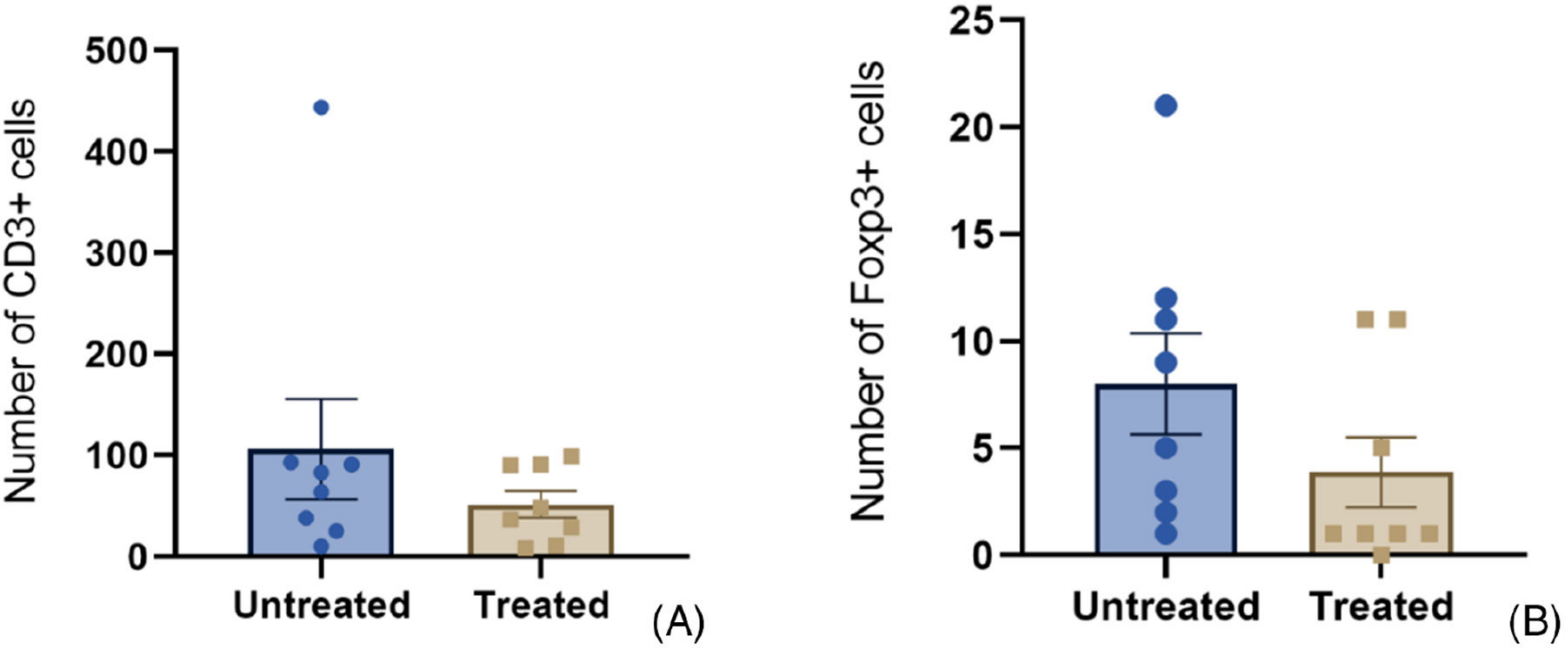
Number of CD3+ (A) and Foxp3+ (B) cells (mean +/− SEM) in sections from untreated areas of the neoplasm compared to counts in sections representing the treatment interface (“Treated”).

Although there were no statistically significant changes, evidence of CD3+ and Foxp3+ cell infiltration was numerically lower in sections from the treatment interface when compared to the untreated sections (*p* = 0.97 and 0.23, respectively, by Kolmogorov–Smirnov test; Figure 5).

**FIGURE 6:**
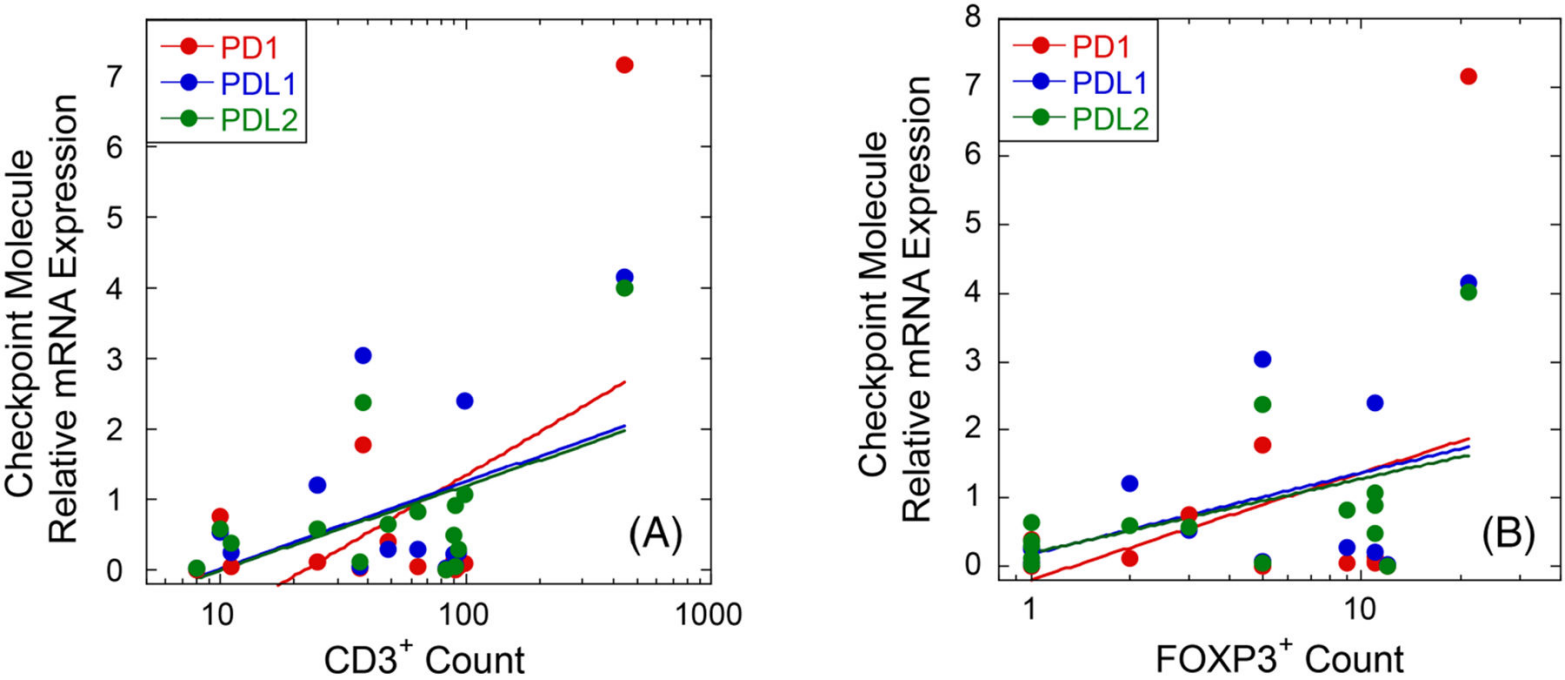
Positive relationship between infiltration of CD3+ (A), *p* = 0.005, *r* = 0.78) and Foxp3+ (B), and relative gene expression of PD-axis. (CD3/PD-1, R^2^ = 0.79, *p* < 0.0001, *r* = 0.89; CD3/PD-L1, R^2^ = 0.44, *p* = 0.006, *r* = 0.66 and CD3/PD-L2; R^2^ = 0.62). (FoxP3/PD-1, R^2^ = 0.64, *p* < 0.01, *r* = 064; CD3/PD-L1, R^2^ = 0.30, *p* = 0.03, *r* = 0.55 and CD3/PD-L2; R^2^ = 0.41, *p* = 0.009, *r* = 0.64). For illustration, curves shown are fitted logarithmically, whereas regression analysis was performed on linear values.

Analysis of covariance was done to assess the relationship between the expression of checkpoint molecules and the infiltration of CD3 and Foxp3 positive cells. All six curves showed positive correlation coefficient (r) values, indicative of a positive relationship (i.e., regression curves with non-zero slope) between the expression of these checkpoint molecules and the infiltration of both CD3 and Foxp3 positive cells (Figure 6).

## 4 ∣ DISCUSSION

The present study evaluated the gene expression of checkpoint molecules from the PD-axis (PD-1, PD-L1, and PD-L2) from 2 cohorts of FFPE tissue sections. The first group, obtained from ViTALS archives, comprised tissue sections from STS grades 1, 2, and 3, in which we predicted an increase in the expression of checkpoint molecules in tumours of higher grades. Consistent with this prediction, a statistically significant increase in the expression of PD-1 was observed in Grade 3 relative to Grade 1. The expression of PD-L1 was higher in Grade 2 STS when compared to STS of Grade 1, and statistically significantly higher when compared to those of Grade 3. Mean relative expression of PD-L2 rose with tumour grade but did not reach statistical significance, Although this pattern of checkpoint molecule increase was anticipated by our hypothesis, STS of Grade 3 maintained levels of PD-L2 comparable to Grade 2, while PD-L1 returned to levels comparable to Grade 1.

The potential importance of PD-L2 is suggested by the gene expression of PD-axis components among STS with varying degrees of undifferentiation. STS with a higher degree of undifferentiation had numerically higher expressions of PD-1 and PD-L2, whereas expression of PD-L1 remained similar among the 3 undifferentiation classifications. Altogether, these findings suggest potentially different mechanisms that regulate the expression of these two ligands and emphasize the role that PD-L2 could play in malignant neoplasia.

Elevated expression of PD-L2 has been reported in human reproductive cancers,^46^ oesophageal carcinomas,^47^ some soft tissue sarcomas,^48^ osteosarcoma,^49^ among others, and it is associated with tumour cell survival, migration, and resistance to chemotherapy.^22,48,50,51^ PD-L2 does not require a conformational change to bind PD-1, as does PD-L1, with a higher affinity for PD-1 than PD-L1.^52^ Yet, PD-L2 has been less thoroughly investigated than has PD-L1, on which the majority of the published findings are focused. Although the reports available on PD-L2's role in therapy response and prognosis are at times contradictory, more recent studies of human neoplasia associate its expression with a poor prognosis.^51,53-55^ More importantly, the expression of this checkpoint molecule has been overlooked in canine neoplasia, with only a sole report in canine melanoma.^27^ These results show a trend of increase for both the receptor PD-1 and the PD-L2 ligand, which supports previous reports suggesting that soft tissue sarcomas with higher degree of malignancy often have a higher infiltration of immune cells, but still are considered immunosuppressed.^19,21,22^ Nevertheless, more data is needed to support these findings and more thoroughly determine the role that PD-L2 may play in the TME and its association with clinical outcomes in canine neoplasia.

Major efforts have been made to develop different therapeutic approaches to treat cancer. Still, reports on how these therapies affect the immune system and modulate the TME are scarce in the human medical literature, and in some cases suggest adaptation by neoplasms to upregulate the expression of PD-L1 after treatment.^56-58^ The second cohort evaluated in this study were archived pre- and post-treatment tissue sections from a clinical trial of histotripsy in ten canine patients with STS. Tissue sections were collected from the treatment interface and untreated areas of the tumour. We predicted that the expression of checkpoint molecules would be lower on sections obtained from the treatment interface, an expectation that was borne out for the three checkpoint molecules analysed.

In the IHC analysis, lower numbers of CD3+ and Foxp3+ cells were observed in tissue sections taken from untreated areas of the neoplasm. Additionally, our IHC analysis shows a positive relationship between the infiltration of CD3+ and Foxp3+ cells with the expression of checkpoint molecules from the PD-axis, as previously reported in canine melanoma.^27^ Evaluation of IBA1+/CD206+ (proinflammatory) macrophages was performed by Ruger et al., reporting intensification of these cells towards the treated area in sections taken from the treatment interface.^40^ The authors also reported that macrophages at both the treatment interphase and untreated areas were devoid of iNOS, usually associated with high expression of PD-L1 through the action of cytokines such as Tumour Necrosis Factor-alpha.^59-61^ Considering these findings, it is possible that the timeline of the study favoured the evaluation of the innate rather than adaptive response. However, the focal decrease of the expression of the PD-axis would promote future infiltration of lymphocytes.

It is important to note that PD-L1 can be expressed by cells other than cancer cells, such as dendritic cells and macrophages, as part of their normal immune homeostatic activity. On the other hand, PD-L2 can be expressed by macrophages, the tumour cells, and the stroma at the TME.^50,52^ The lack of commercially-available antibodies for these checkpoint molecules for canine neoplasms has limited our ability to localize PD axis molecules within the TME, and thus further studies are needed to determine PD-L2 expression and assess its contribution to malignant features of the tumour. However, the higher expression of checkpoint molecules in STS of higher grades suggests an important role in this process that could be further studied by follow-up of these patients that undergo histotripsy, as its higher expression is usually associated with shorter survival times and poor prognosis. The presence of CD3+ lymphocytes in the untreated areas, along with the expression of the PD-axis, makes these patients potential candidates for combined therapy with histotripsy and anti-PD-1 immunotherapy.

## Supplementary Material

Figure S1. Histotripsy treatment interface. Example of tumour tissue sampled immediately adjacent to the area of ablation, as identified by evidence of coagulation necrosis of tumour tissue induced by the treatment. Table S1. Histotypes of tumours used.

## Data Availability

The data that support the findings of this study are available from the corresponding author upon reasonable request.
