# Supplementary material for: Inhibitory checkpoint molecule mRNA expression in canine soft tissue sarcoma
Source: Vet Comp Oncol. Author manuscript; Available in PMC 2024 Feb 5. (PMC10841275; doi:10.1111/vco.12934)

Supplementary Material for VCO-2023-030 R1: “Inhibitory Checkpoint Molecule mRNA Expression in Canine Soft Tissue Sarcoma”

**Conditions for Immunohistochemistry**

Immunohistochemical staining was performed using a Ventana Benchmark XT automated stainer. Tissue sections were deparaffinized with xylene and rehydrated through a graded series of alcohols. Antigen retrieval was conducted by incubation in ULTRA Cell Conditioning Solution (Ventana/Roche 950-224) for 64 min at 95C. Conditioned sections were incubated with primary antibody for 32 min at 36C, followed by incubation with secondary antibody (ultraView Universal Alkaline Phosphatase Red Detection Kit, Ventana/Roche 760-501) for 16 min at the same temperature. Reporter color was developed using Red Naphthol and Fast Red chromagen for 16 min per the ultraView Universal AP kit protocol. Sections were counterstained with hematoxylin and coverslipped.

**Table S1. Histotypes of tumors used**

| **Tumor Histotype** | **Archival STS Case Study** | | | **Histotripsy Study** |
| --- | --- | --- | --- | --- |
|  | **Grade 1** | **Grade 2** | **Grade 3** |  |
| Perivascular wall tumor | 8 | 5 | - | 4 |
| Peripheral nerve sheath tumor | 2 | 2 | - | 2 |
| Soft tissue sarcoma without identifiable histotype (STS) | - | - | 7 | 1 |
| Myxosarcoma | - | 3 | - | 2 |
| Fibrosarcoma | - | - | - | 1 |
| Anaplastic STS | - | - | 1 | - |
| Perivascular wall tumor/STS | - | - | 1 | - |

**Figure S1. Histotripsy treatment interface.** Example of tumor tissue sampled immediately adjacent to the area of ablation, as identified by evidence of coagulation necrosis of tumor tissue induced by the treatment.


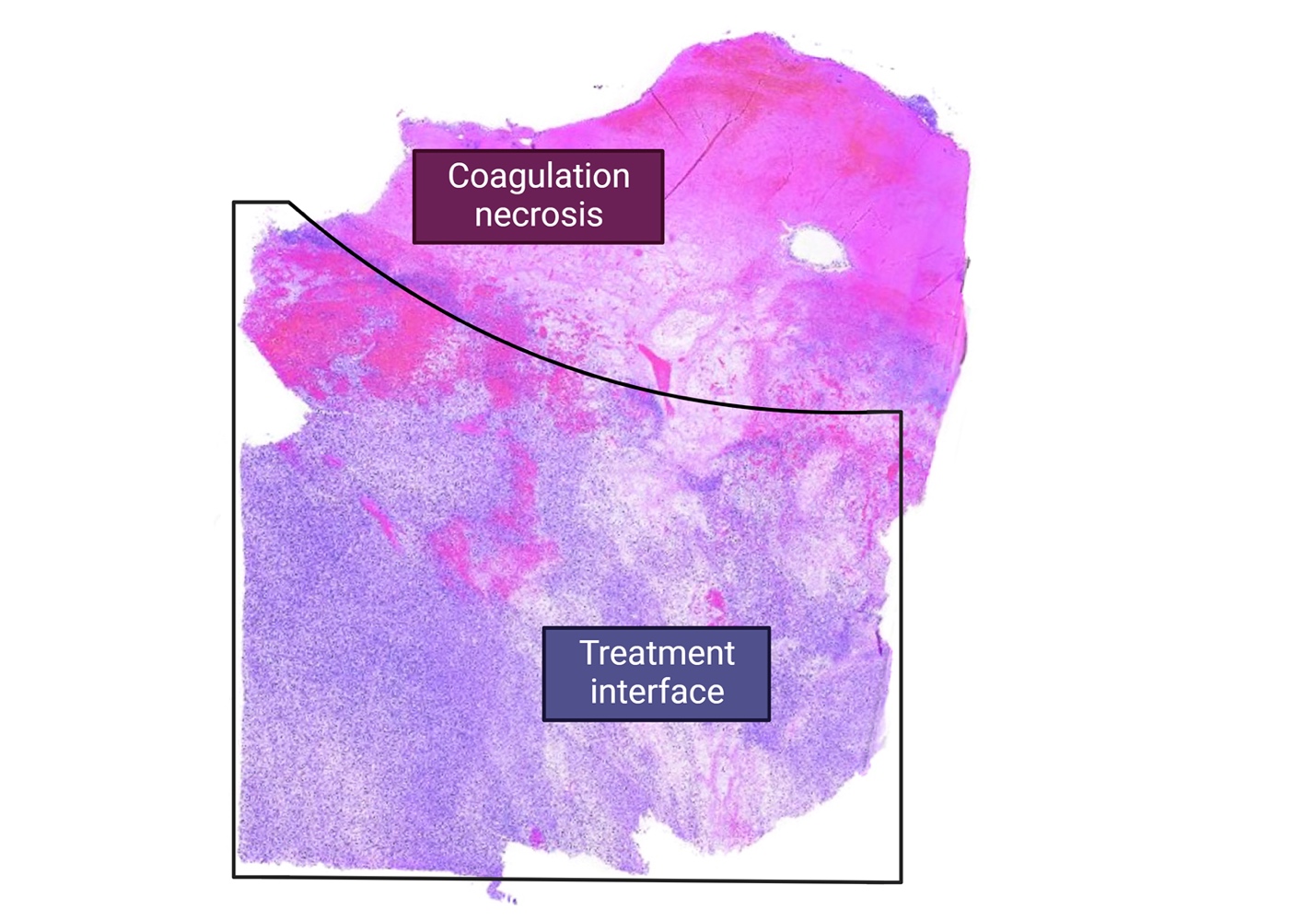

Supplement: Figure S1. Histotripsy treatment interface. Example of tumour tissue sampled immediately adjacent to the area of ablation, as identified by evidence of coagulation necrosis of tumour tissue induced by the treatment. Table S1. Histotypes of tumours used. [file NIHMS1931103-supplement-Figure_S1__Histotripsy_treatment_interface__Example_of_tumour_tissue_sampled_immediately_adjacent_to_the_area_of_ablation__as_identified_by_evidence_of_coagulation_necrosis_of_tumour_tissue_induced_by_the_treatment___T.docx]
